# Supplementary material for: Simple Detection of DNA Methyltransferase with an Integrated Padlock Probe
Source: Biosensors (Basel). 2022 Jul 26;12(8):569. doi: 10.3390/bios12080569 (PMC9332213; doi:10.3390/bios12080569)
Supplement: Supplementary file 1 [file biosensors-12-00569-s001.zip › biosensors-1778853-supplementary.pdf]

Supporting Information

Simple detection of DNA methyltransferase with an integrated padlock probe

Yuehua Wang [1,2], Yingli Han [1,2], Fangyu Zhou [1], Tingting Fan [1] and Feng Liu [1,2,*]

[1] State Key Laboratory of Chemical Oncogenomics, Guangdong Provincial Key Laboratory of Chemical Biology, Tsinghua Shenzhen International Graduate School, Shenzhen, 518055, PR China.

[2] Department of Chemistry, Tsinghua University, Beijing, 100084, PR China.

***** Correspondence: liu.feng@sz.tsinghua.edu.cn; Tel./Fax.: +86-0755-26036533

Table S1. Oligonucleotides used in this study.

Name	Sequence(5'-3')
PPs	5'OH-TA**CCGG**ATCAGACTGATGTTGA-3'OH
PPs M	5'OH-TAC*m5*CGGATCAGACTGATGTTGA-3'OH
PPc	5'P-TGAT**CCGG**TAGTTTCATCAAAGCCCATACTACAACCTCAACATCAGTC-3'OH

Annotation: bold part is the site of M.SssI and HpaII, tilt part is the 5-methylcytosine, red part of PPc is the complementation part with PPs

Table S2. Detection limits and real sample applications of some MTase detection methods.

Method	**Linear range(U/mL)**	**Detection limit(U/mL)**	**Strategy of amplification**	**Real sample**	**Recovery rate (%)**	**Ref.**
Colorimetric	0.08-50	0.069	NO	Cell lysates of A549	97.0-101.7	[1]
fluorescence	2.5-70	1.8	HRCA	10% human serum samples	97.0-104.0	[2]
Fluorescence	0.1-20	0.05	NO	1% serum samples	97-98.9	[3]
Fluorescence	0-1	0.015	HCR	NO		[4]
Fluorescence	0.01-100	0.01	SDA	NO		[5]
electrochemical	0.001-1	0.0005	NO	10% human serum samples	97.6-105.0	[6]
chemiluminescent immunoassay	0.0082	0.01-30	NO	NO		[7]
surface-enhanced Raman scattering (SERS)	0.05-50	2.8×10^{-3}	RCA	10% human serum samples	99.6-107	[8]
Fluorescence	0.5-110	0.0404	RCA	10% human serum samples	96.09-103.5	This work

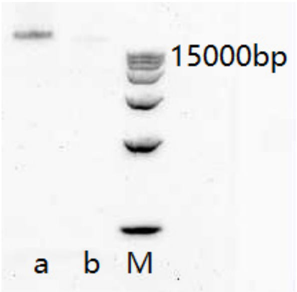

Figure S1. Electrophoresis analysis of the products of RCA of the detection system with (Lane a) and without (Lane b) M.SssI by RCA .(Lane M) 15000 bp DNA markerI.

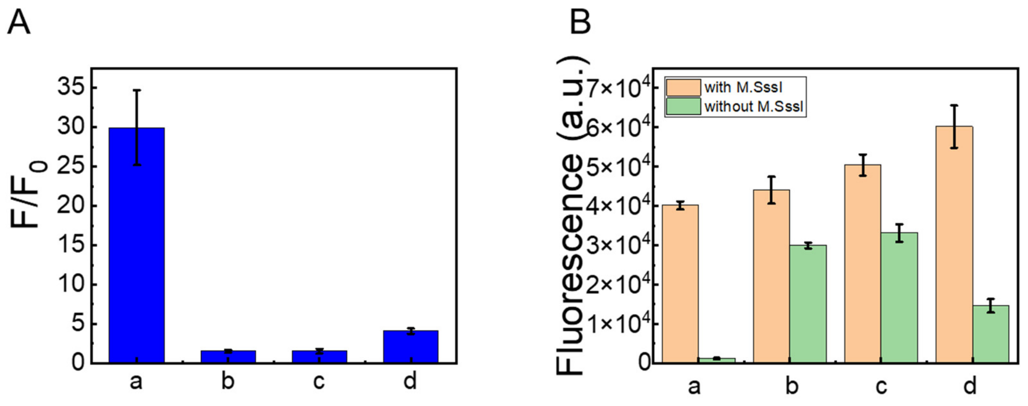

Figure S2. Optimize the methylation and cleavage buffers. (A) F is the fluorescence intensity with M.SssI and F0 is fluorescence intensity without M.SssI. (B) The fluorescence intensities of the sensing systems with or without M.SssI. (a) CutSmart buffer (methylation process) + CutSmart buffer (cleavage process); (b) NEBuffer 2(methylation process) + CutSmart buffer (cleavage process); (c) NEBuffer 2(methylation process) + CutSmart buffer + Lambda reaction buffer (cleavage process); (d) CutSmart buffer (methylation process) + Lambda reaction buffer (cleavage process).

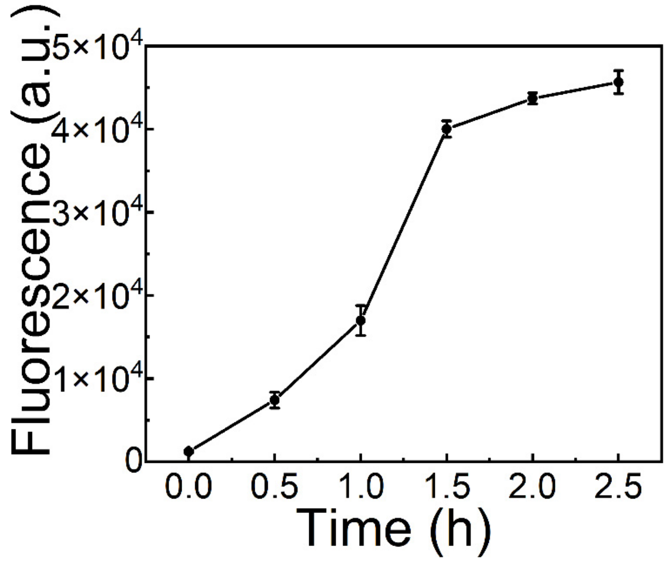

Figure S3. Optimize the time of methylation process.

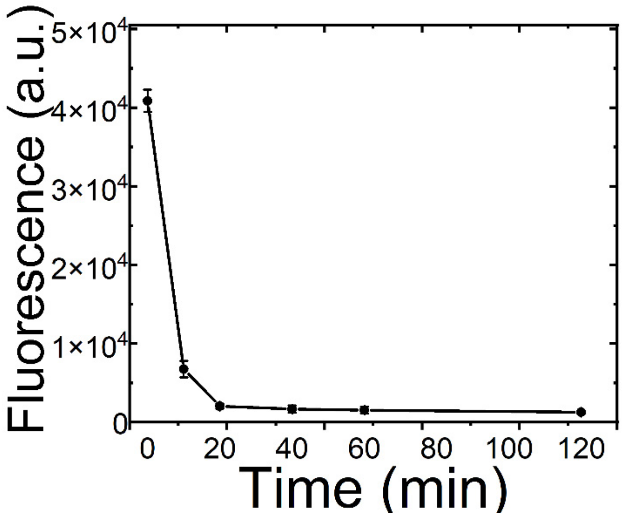

Figure S4. Optimize the time of cleavage of HpaII and Lambda exo.

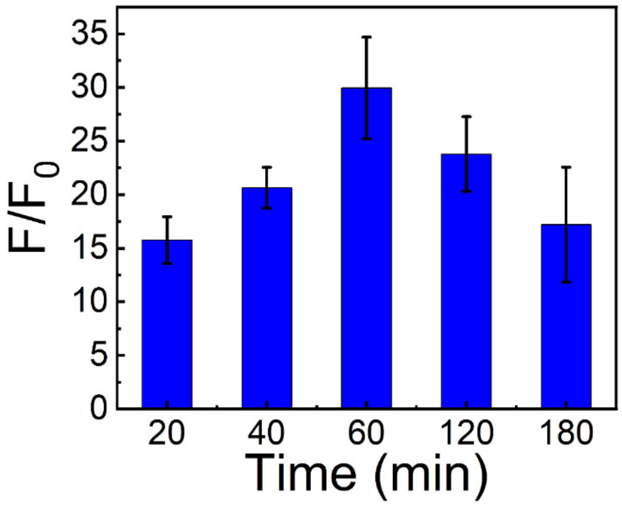

Figure S5. Optimize the time of digestion of ExoI.

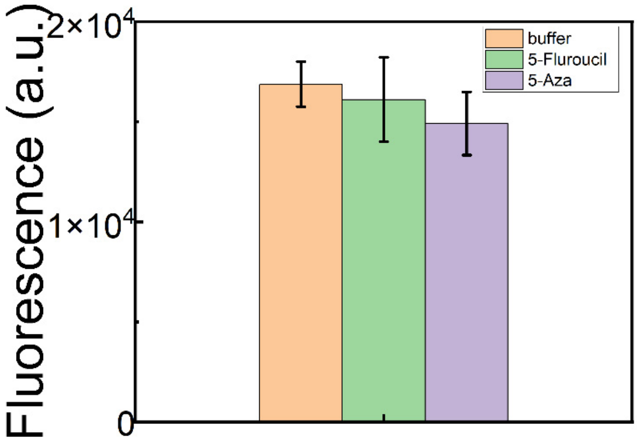

Figure S6. Eliminate the probable impact of detection system. The concentration of M.SssI is 50 U/mL.

References

1. Li, Z.-M.; Zhong, X.-L.; Wen, S.-H.; Zhang, L.; Liang, R.-P.; Qiu, J.-D. Colorimetric detection of methyltransferase activity based on the enhancement of CoOOH nanozyme activity by ssDNA. *Sensors and Actuators B-Chemical* **2019,** *281*, 1073-1079. https://doi.org/10.1016/j.snb.2018.11.085
2. Chen, L.; Zhang, Y.; Xia, Q.; Luo, F.; Guo, L.; Qiu, B.; Lin, Z. Fluorescence biosensor for DNA methyltransferase activity and related inhibitor detection based on methylation-sensitive cleavage primer triggered hyperbranched rolling circle ampli fication. *Analytica Chimica Acta* **2020,** *1122*, 1-8. https://doi.org/10.1016/j.aca.2020.04.061
3. Dadmehr, M.; Karimi, M. A.; Korouzhdehi, B. A signal-on fluorescence based biosensing platform for highly sensitive detection of DNA methyltransferase enzyme activity and inhibition. *Spectrochimica Acta Part a-Molecular and Biomolecular Spectroscopy* **2020,** *228*, https://doi.org/10.1016/j.saa.2019.117731
4. Wang, Q.; Pan, M.; Wei, J.; Liu, X.; Wang, F. Evaluation of DNA Methyltransferase Activity and Inhibition via Isothermal Enzyme-Free Concatenated Hybridization Chain Reaction. *Acs Sensors* **2017,** *2* (7), 932-939. https://doi.org/10.1021/acssensors.7b00168
5. Chen, S.; Ma, H.; Li, W.; Nie, Z.; Yao, S. An entropy-driven signal amplifying strategy for real-time monitoring of DNA methylation process and high-throughput screening of methyltransferase inhibitors. *Analytica Chimica Acta* **2017,** *970*, 57-63. https://doi.org/10.1016/j.aca.2017.03.017
6. Tian, R.; Liu, D. Weng, T.; Yin, Y.; Xie, W.; Yin, B.; Shi, B.; Tlili, C.; Wang, D. DNA-functionalized biosensor for amplifying signal detection of DNA methyltransferase activity. *Journal of Electroanalytical Chemistry* **2021,** *891*, https://doi.org/10.1016/j.jelechem.2021.115260
7. Yan, X.-L.; Xue, X.-X.; Deng, X.-M.; Jian, Y.-T.; Luo, J.; Jiang, M.-M.; Zheng, X.-J. Chemiluminescence strategy induced by HRP-sandwich structure based on strand displacement for sensitive detection of DNA methyltransferase. *Microchemical Journal* **2020,** *158,* https://doi.org/10.1016/j.microc.2020.105183
8. Chen, R.; Shi, H.; Meng, X.; Su, Y.; Wang, H.; He, Y. Dual-Amplification Strategy-Based SERS Chip for Sensitive and Reproducible Detection of DNA Methyltransferase Activity in Human Serum. *Analytical Chemistry* **2019,** *91* (5), 3597-3603. https://doi.org/10.1021/acs.analchem.8b05595
